# Cytostatic and Anti-tumor Potential of Ajwa Date Pulp against Human Hepatocellular Carcinoma HepG2 Cells

**DOI:** 10.1038/s41598-018-36475-0

**Published:** 2019-01-21

**Authors:** Sahabjada Siddiqui, Rumana Ahmad, Mohsin Ali Khan, Shivbrat Upadhyay, Ishrat Husain, Anand Narain Srivastava

**Affiliations:** 1 Department of Biotechnology, Era’s Lucknow Medical College & Hospital, Era University, Lucknow, 226003 UP India; 2 Department of Biochemistry, Era’s Lucknow Medical College & Hospital, Era University, Lucknow, 226003 UP India; 3 Chancellor, Era University, Lucknow, 226003 UP India; 4 Department of Pathology, Era’s Lucknow Medical College & Hospital, Era University, Lucknow, 226003 UP India

## Abstract

Ajwa dates (*Phoenix dactylifera* L.) are used by traditional therapeutic practitioners for several health benefits but most remain to be scientifically validated. In this study, we evaluated the apoptosis-inducing effect of ethanolic extract of Ajwa date pulp (ADP) on human hepatocellular carcinoma (HCC) HepG2 cells. High performance liquid chromatography analysis revealed the presence of polysaccharide β-D-glucan in ADP extract. Treated HCC cells revealed morphological characteristics of apoptosis under phase contrast microscopy. MTT assay demonstrated significant (*p* < 0.05) dose- and time-dependent inhibition of HCC cell growth. HCC cells were found to be in late apoptotic stage on treatment with higher doses of ADP extract as depicted by acridine orange/ethidium bromide and Annexin V-FITC/PI double stain. Importantly, ADP extract increased the reactive oxygen species level and decreased the mitochondrial membrane potential in treated HCC cells. Flow cytometry analysis demonstrated that ADP extract induced elevation of S and G2/M phases of cell cycle. Moreover, ADP extract induced apoptosis in HCC cells independent of tumor suppressor genes *viz*. CHEK2, ATM and TP53. Interestingly, ADP extract did not display any significant effect on normal cell line Vero. This study provides validation that ADP extract can be considered as a safe and natural potential drug candidate against human liver cancer.

## Introduction

Cancer is a rapidly growing health problem around the world, caused mainly due to mutation, adverse environmental conditions, dietary habits, and lifestyle^1,2^. Amongst cancer, hepatocellular carcinoma (HCC) is the second most common cause of cancer death. HCC is the fifth most common malignant tumor of the liver in adults and occurs predominantly in patients with underlying chronic liver disease and cirrhosis^3^. Due to HCC, there are approximately 800,000 deaths per annum globally^4^. The prevalence of HCC is increasing gradually in the world during the past decade^5^.

Various treatment strategies based on hepatic resection, orthotopic liver transplantation, chemo-embolization and systemic chemotherapies^6^, are available. Amongst all treatment managements, chemotherapy is the main therapeutic method for advanced HCC. However, drug resistance and side-effects of liver failure accompanying the disease limit the successful outcome in most cases^7^. In this context, natural products may be considered as safe and effective alternative therapeutic methods for the treatment of HCC and hence, it is essential to rigorously explore novel natural products that effectively cure HCC patients.

Ethno-traditional uses of plant-derived natural products have been a major source for the discovery of potential anticancer agents^8^. Natural products are primary sources of effective anticancer drugs with novel structures and unique mechanisms of action for the treatment of various forms of cancer^9^. Various plant-derived natural products such as alkaloids, flavonoids, polysaccharides, saponins, and terpenes have been used against HCC^10^. However, most of the plant-derived natural products are toxic or ineffective which limit the use of natural products for drug discovery. Therefore, there is a necessity for the pursuit of novel plant-derived products that have potential against cancer cells as well as relatively little or no toxicity towards the normal cells.

Ajwa dates (*Phoenix dactylifera* L.) are one of the most popular natural fruits that belong to the holy city of Al-Madinah Al-Munawara and its neighboring areas in the Kingdom of Saudi Arabia (KSA). Ajwa date has been described in the traditional and alternative medicine to provide several health benefits including anticholesteremic, antidiabetic, anti-inflammatory, antioxidant, hepatoprotective and anticancer effects^11,12^. The previous phytochemical investigations have revealed that Ajwa date pulp (ADP) contains approximately 80% reducing sugars mostly fructose, glucose, galactose, and maltose along with various flavonoids, glycosides, polyphenols, and phytosterols^11,13–15^. Phytochemicals present in Ajwa fruits exhibit anti-inflammatory, antioxidant, cardioprotective, hypolipidemic and anti-apoptotic properties^16^. A previous study has reported that the aqueous extract of Ajwa dates inhibits diethylnitrosamine-induced liver carcinoma in a rat model^17^. Similarly, methanolic extract of Ajwa dates has been reported to inhibit the growth of human breast cancer MCF7 cells and ethyl acetate extract of Ajwa dates has been found to reduce the growth of prostate cancer PC3 cells by causing cell cycle arrest^18,19^. Remarkably, no work has been done so far to explore the *in vitro* apoptosis-inducing mechanism of cell death of Ajwa dates on HepG2 cell line.

The present study describes the effects of Ajwa dates against HCC cells. High performance liquid chromatography (HPLC) analysis was also carried out to identify the bioactive components in ADP extract. The study was subjected to several parameters in order to analyze the apoptosis-inducing effects *via* ROS generation, regulation of cell cycle arrest and modulation of expression of tumor suppressor genes *viz*. checkpoint kinase 2 (CHEK2), ataxia telangiectasia (A-T) mutated (ATM) protein kinase, and tumor protein 53 (TP53). Therefore, the present study makes significant contributions to the field of natural-product based therapeutics in cancer.

## Materials and Methods

### Reagents and chemicals

Dulbecco’s Modified Eagle Medium Nutrient Mixture F-12 (DMEM/F-12), fetal bovine serum (FBS), antibiotic penicillin and streptomycin solution and β-D-glucan powder were purchased from Sigma-Aldrich, USA. Hoechst 33342, propidium iodide (PI), Ribonuclease A (RNase A), 2,7-dichlorodihydrofluorescein diacetate (DCFH-DA), acridine orange (AO), ethidium bromide (EtBr) and rhodamine 123 (Rh 123) were purchased from Himedia, India. All other chemicals and reagents used were of analytical grade.

### Preparation of Ajwa date extract

Fresh Ajwa dates were procured from Al-Madina Al-Munawwarah, Kingdom of Saudi Arabia. The pulp part of date fruits was manually separated, washed with double distilled water, oven dried and coarsely powdered using pestle and mortar. The coarse powder contents were then extracted in 95% ethanol (1:3 ratio, weight to volume) at 25 °C for 3 days. The extracted solvents were pooled and filtered through Whatman No. 1 filter paper (125 mm). The filtrate obtained was concentrated in vacuum at 45 °C using Rotavapor (Buchi Rotavapor R-205, Switzerland). The obtained extract was further concentrated in a water bath until a semi-solid paste was obtained and stored in an air-tight container until further use in experiments.

### HPLC analysis of ADP extract

ADP extract was characterized using HPLC on a Waters 515 HPLC Pump system (Milford, USA) equipped with a Waters 2998 PDA detector, a Waters column temperature controller, a pump control module coupled with an empower chromatography workstation. For chromatographic analysis, an XBridge C_18_ Reverse Phase column (4.6 × 250 mm, 5 μm), with gradient elution as the mobile phase, was adopted. The mobile phase consisted of a mixture of water (Solvent A) and methanol-acetonitrile (Solvent B) which was applied as a gradient for 40 min. The standard β-D-glucan and ADP extract were dissolved in HPLC grade water and filtered through a 0.45 μm membrane filter and the injection volume was 10 μL. The flow rate was 1.0 mL/min and the column temperature was set at 30 °C. The gradient of mobile phase was as follows: 80% A, 20% B for 0–6 min; 70% A, 30% B for 6–12 min, 30% A, 70% B for 12–18 min; 20% A, 80% B for 18–25 min; 20% A, 80% B for 25–30 min; 80% A, 20% B for 30–35 min and 90% A, 10% B for 35–40 min. HPLC was monitored at 254 nm to provide real-time chromatograms of both standard and ADP extract.

### Cell lines and culture

Human HCC HepG2 and normal kidney epithelial Vero cell lines were obtained from the cell repository of National Centre for Cell Sciences, Pune, India. Cells were grown in Dulbecco’s Modified Eagle Medium: F12 (1:1) supplemented with 10% heat-inactivated Fetal Bovine Serum, 2 mM L-glutamine, 1% penicillin and streptomycin solution. Cells were cultured in cell culture flasks and were kept in an incubator (Thermo Scientific, USA) at 37 °C and 5% CO_2_.

### MTT assay

The antiproliferative activity of ADP extract was evaluated by MTT reduction assay following a previously published protocol^20^. HepG2 and Vero cells were seeded at a density 1 × 10^4^ cells/mL in 96-well microtiter culture plates and incubated overnight. ADP extract was diluted in culture media and treated in triplicate with different concentrations (10, 15, 20, 25 and 30 mg/mL) of ADP extract for 24 and 48 h. The absorbance values were read in an ELISA plate reader (Biorad-PW41, USA) at 550 nm with a reference wavelength of 630 nm. The cellular morphological changes were observed under an inverted phase contrast microscope (Nikon Eclipse TS100, Japan).

### Nuclear condensation assay

Based on the cell viability assay, the apoptosis-inducing effect of ADP extract was evaluated at two effective doses *viz*. 15 and 25 mg/mL. DNA condensation was measured using Hoechst 33258 staining as per a previously published method^21^. To assess nuclear morphology, stained cells were captured under an inverted fluorescence phase contrast microscope (Zeiss AxioVert 135, USA).

### Acridine orange-ethidium bromide (AO/EtBr) assay

The mechanism of cytotoxicity of ADP extract on HepG2 cells was evaluated as reported previously^22^. HepG2 cells were seeded in a 24-well culture plate and treated at 15 and 25 mg/mL of ADP extract for 48 h. The cells were stained with AO/EtBr (2 µg/mL each) fluorescent dyes for 10 min at 37 °C in a CO_2_ incubator. Subsequently, cells were washed twice with ice-cold phosphate buffer saline (PBS) and observed under an inverted fluorescence phase contrast microscope (Zeiss AxioVert 135, US).

### DNA fragmentation assay

Genomic DNA was isolated from both treated and untreated cells as per instruction manual of NucleoSpin^®^ Blood Kit (Macherey-Nagel, Germany). Briefly, HepG2 cells at a density 1 × 10^6^ were cultured in T-25 cm^2^ culture flasks overnight and cells were then treated with different concentrations of ADP extract for 48 h. Treated cells were washed with PBS and resuspended in 200 µl PBS. Electrophoresis of extracted DNA was performed on 1.5% agarose gel at 60 V for 120 min using 1x TBE buffer in a gel electrophoresis unit (Genei, India). DNA bands were observed under ultraviolet illumination gel-doc system (BIORAD, USA).

### Analysis of apoptosis by Annexin V-FITC double stain

Apoptotic cells were quantified using an Annexin V-FITC Apoptosis Kit (BioVision, USA) according to manufacturer’s protocol by flow cytometry. Briefly, cells at 1 × 10^6^ cells/mL density were incubated with 15 and 25 mg/mL concentrations of ADP extract for 48 h. Cells were then harvested and re-suspended in the binding buffer and stained with 2 μl Annexin V-FITC and 2 μl PI for 15 min at 25 °C in the dark. The apoptotic index was immediately analyzed by flow cytometry (FACS Canto II flow cytometer, BD Biosciences, USA).

### Intracellular reactive oxygen species (ROS) measurement

Intracellular ROS levels were estimated using DCFH-DA dye by fluorescence microscopy imaging and flow cytometry techniques as reported previously^20^. Intracellular fluorescence intensity of cells was visualized under an inverted fluorescence microscope. To quantify ROS intensity, both treated and untreated cells were harvested and washed with PBS and incubated in PBS containing 10 µM DCFH-DA dye at 37 °C for 20 min. The cells were then washed twice with PBS and subjected to flow cytometry analysis.

### Mitochondrial membrane potential (MMP, ΔΨm) measurement

The alterations in MMP were assessed with the fluorescent probe Rh 123 as per previously published protocol^23^. The images of incubated cells were captured under a fluorescence microscope. For flow cytometry analysis, treated cells were incubated with Rh 123 at a final concentration of 10 µM for 30 min in dark. After washing with PBS twice, cells were resuspended in 500 µl PBS and analyzed using flow cytometry.

### Analysis of cellular DNA content

Cells were seeded at density 1 × 10^6^ cells/mL into 6-well plates and treated with ADP extract (15 and 25 mg/mL) for 48 h. Different phases of the cell cycle with cellular DNA contents were analyzed using flow cytometry as described previously^20^.

### Real-time quantitative-PCR (qRT-PCR)

HepG2 cells were cultured in T-25 cm^2^ culture flasks and treated with 15 and 25 mg/mL of ADP extract for 48 h. After the desired treatment periods, cells were lysed with lysis buffer and pooled into 15 ml tubes. Purified total RNA was eluted using the PureLink™ RNA Micro Kit (Invitrogen, USA) according to manufacturer’s instructions. Eluted RNA was resuspended in RNase-free water and quantified with a Nanodrop (Thermo Scientific, USA). First-strand cDNA was synthesized using the SuperScript^™^ VILO^™^ cDNA Synthesis Kit as per manufacturer’s protocol (Invitrogen, USA). Quantitative real-time PCR analysis was performed using Applied Biosystems TaqMan^®^ Fast Advanced Master Mix and TaqMan^®^ Gene Expression Assays in a real-time PCR machine (Applied Biosystems StepOnePlus system version 2.3, Canada). All data were then normalized to GAPDH which was used as an endogenous control. Fold change in gene expression was calculated using the comparative threshold cycle (∆∆CT) method. The IDs for TaqMan Gene Expression Assays used in this experiment are listed in Table 1.

**Table 1:** IDs for TaqMan gene expression assays.

Target gene	Assay ID
GAPDH	Hs02786624
CHEK2	Hs00200485
ATM	Hs00175892
TP53	Hs01034249

### Statistical analysis

Cell viability data were expressed as the mean ± SEM from three independent experiments. Statistical evaluation was determined by one-way ANOVA followed by Dunnett’s Multiple Comparison Test using GraphPad Prism software (Version 5.01). A *p*-value less than 0.05 was considered as statistically significant.

## Results

### HPLC characterization of ADP extract

**Figure 1:**
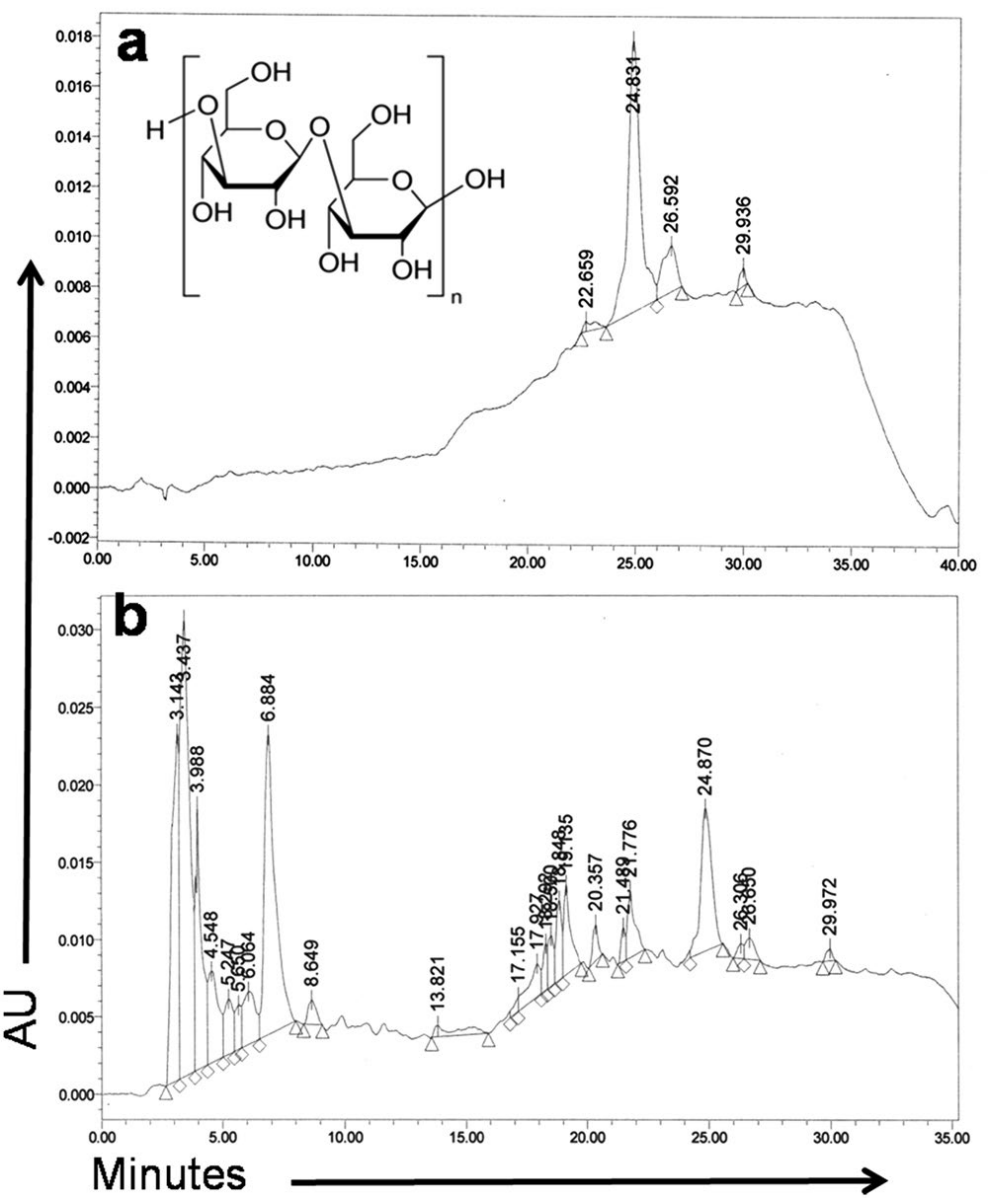
HPLC profile of standard β-D-glucan and ADP extract (**a**) HPLC chromatogram of β-D-glucan standard (R_t_ = 24.831 min) (**b**) HPLC chromatogram of ADP extract. The optimum peak had an R_t_ of 24.87 min which was similar to that of standard β-D-glucan.

The chromatograms of standard β-D-glucan and ADP extract are presented in Fig. 1. The peak area and percentages of different components with a specific retention time (R_t_) in HPLC chromatograms are shown in Table S1. The HPLC chromatographic analysis using a reverse phase column and water (A) and methanol-acetonitrile (B) solvents as mobile phase provided fine separation of β-D-glucan with R_t_ value of 24.831 min at 254 nm in chromatograms (Fig. 1a and Table S1). The corresponding peak of ADP extract in HPLC analysis was found at a R_t_ value of 24.87 min under similar conditions (Fig. 1b). This study revealed the presence of β-D-glucan as an active component in ethanolic extract of ADP.

### Effect of ADP extract on morphological changes and cell viability of HCC cells

**Figure 2:**
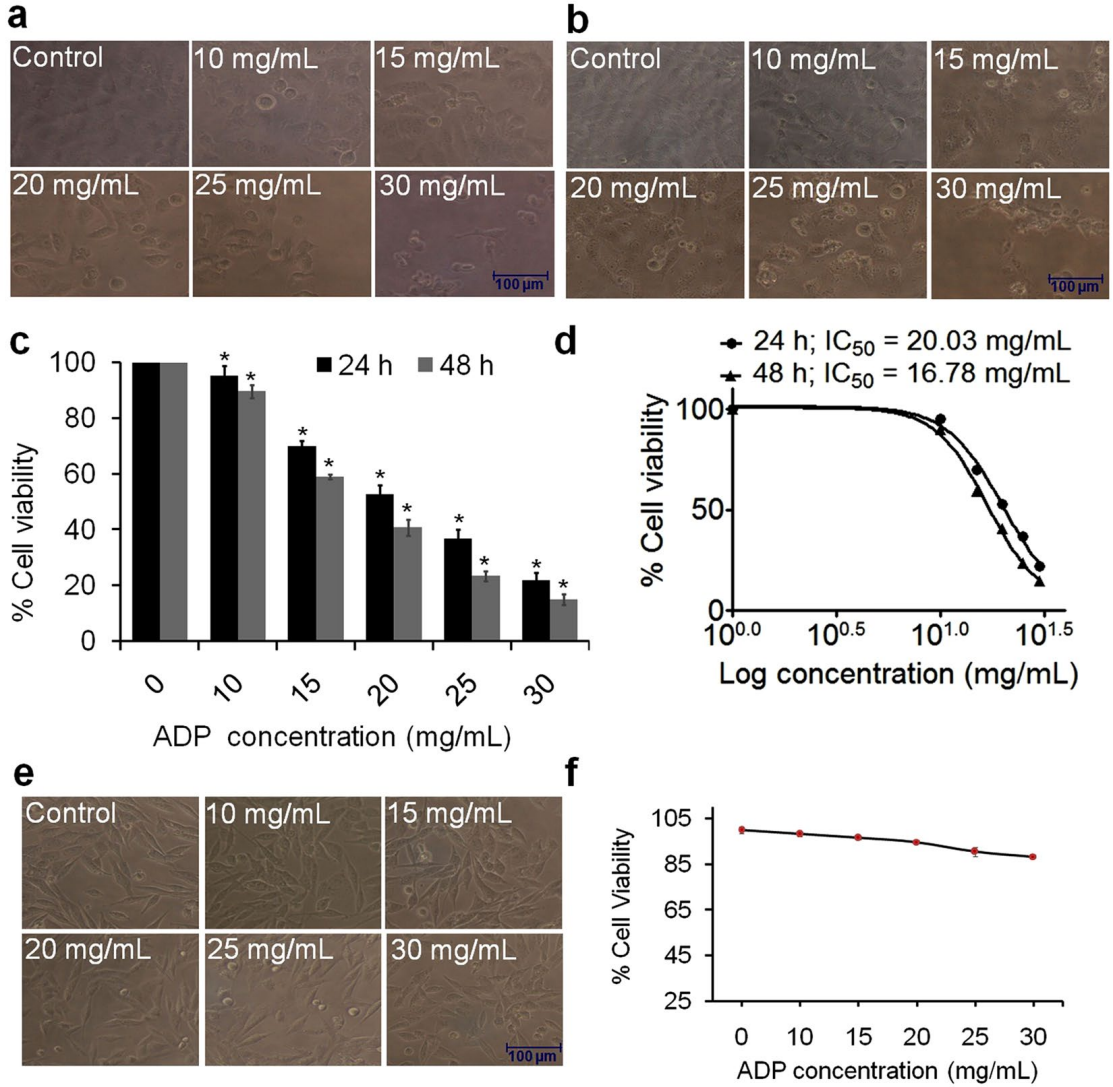
Microscopic observation and cytotoxic activity of different concentrations (10–30 mg/mL) of ADP extract against HCC and normal Vero cells (**a**) & (**b**) Photomicrograph of HepG2 cells treated with 10 to 30 mg/mL concentrations of ADP extract at 24 and 48 h, respectively. Photomicrographs were taken with an inverted phase contrast microscope. Scale bar = 100 μm. (**c**) Percent cell viability of ADP extract at various concentrations on HepG2 cells after 24 and 48 h incubation. (**d**) Dose response curve (Log concentration *vs* % cell viability) representing IC_50_ values of ADP extract at 24 and 48 h incubation. (**e**) Photomicrograph of Vero cells at different concentrations of ADP extract after 24 h. (**f**) Percent cell viability of Vero cells at various concentrations of ADP extract after 24 h incubation. Values are expressed as mean ± SEM of three independent experiments. ^*^p < 0.05 as compared to control.

HCC cells were treated with increasing concentrations of ADP extract and photographed at 24 and 48 h of ADP exposure. Morphological comparison of treated and untreated HCC cells revealed severe ADP-mediated variations. As shown in Fig. 2a, the unexposed cells exhibited normal features such as a typical adherent, homogeneous and even cell surface at both 24 and 48 h of incubation. Following exposure to 10–30 mg/mL ADP extract for 24 h, the majority of HCC cells developed a non-adherent, detached and rounded morphology. Moreover, ADP extract increased the drastic morphological changes in HCC cells at 48 h incubation periods, with typical apoptotic features thus exhibiting both dose-and time-dependent prevalence and severity (Fig. 2b). As indicated in Fig. 2c, at 24 h treatment period, ADP extract reduced cell viability to 95.3, 70, 52.7, 36.6 and 22% at 10, 15, 20, 25 and 30 mg/mL, respectively, of the extract concentrations. Conversely, ADP extract exerted a more pronounced effect at 48 h, drastically reducing the viability of HCC treated cells to 89.6, 58.9, 40.8, 23.4 and 15% at 10, 15, 20, 25 and 30 mg/mL of ADP extract, respectively. Thus, the cell viability data suggested that ADP treatment significantly reduced HCC cell growth in both dose- and time-dependent manner, indicating its ability to impair proliferation potential. ADP extract reduced HCC cell number with an IC_50_ value of 20.03 and 16.78 mg/mL after 24 and 48 h exposure, respectively (Fig. 2d). Moreover, Fig. 2e represents the cellular morphology of normal Vero cell line and Fig. 2f represents the corresponding percent cell viability at 10, 15, 20, 25 and 30 mg/mL concentrations of ADP extract after 24 h incubation period. ADP extract showed low toxicity against Vero cells and the percent cell survival was found to be 98.4, 96.6 and 94.8% at 10, 15 and 20 mg/mL concentrations of ADP extract. Moreover, 90.5 and 88.4% survival was observed at 25 and 30 mg/mL, respectively. These results suggest that ADP extract did not show any significant changes in morphology and survival of normal cell line Vero.

### ADP extract stimulated chromatin condensation and induced apoptosis

**Figure 3:**
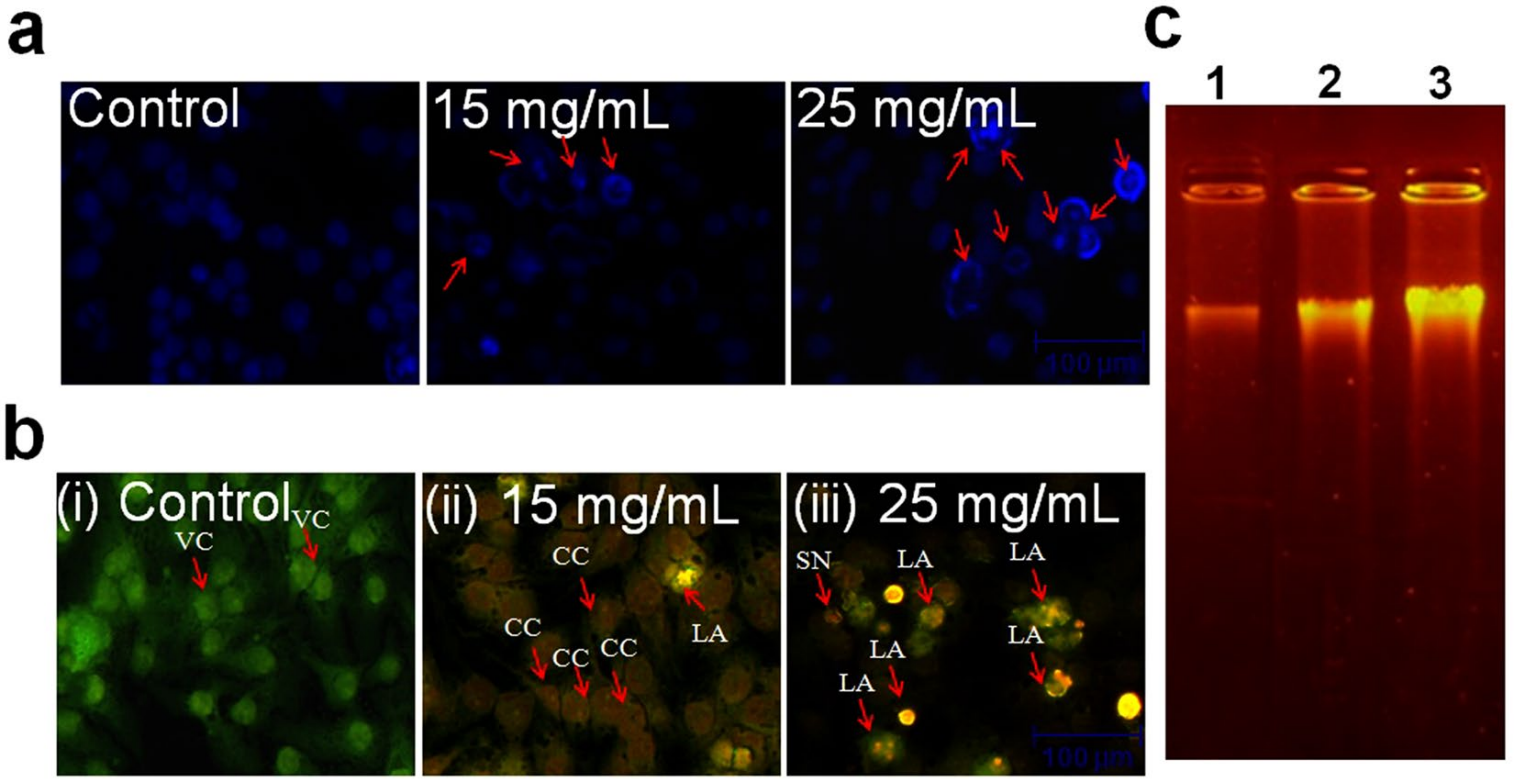
Apoptosis-inducing activity of ADP extract in HCC treated cells (**a**) Chromatic condensation of HCC treated cells at 15 and 25 mg/mL of ADP extract after 48 h. (**b**) Fluorescent micrographs of AO/PI-double-stained HCC cells at 15 and 25 mg/mL of ADP extract after 48 h. (i) Untreated HepG2 cells depict healthy structure (ii) Early apoptosis features such as chromatin condensation and membrane blebbing were observed at 15 mg/mL of ADP extract (iii) Late apoptosis and secondary necrosis were observed at 25 mg/mL of extract. VC: Viable cells; CC: Chromatin condensation; LA: Late apoptosis and SN: Secondary necrosis. Scale bar = 100 μm. (**c**) DNA fragmentation assay in HCC cells as an index of apoptosis. Lane 1: showing control HepG2 cells; Lane 2, and 3: cells treated with 15 and 25 mg/mL of ADP extract, respectively.

As is evident from photomicrograph (Fig. 3a), ADP extract at 15 mg/mL increased the chromatin condensation in HCC cells as compared to control. However, 25 mg/mL of ADP extract exhibited maximum nuclear condensation. Furthermore, the AO/EtBr double stain revealed that control cells displayed uniformly stained green-colored nuclei indicating live and healthy cells. Treated cells appeared either green-colored with condensed nuclei indicative of early apoptosis, or orange-red colored cells with condensed nuclei indicative of late apoptosis. HCC cells displayed early apoptotic features at low doses whereas late apoptotic features were observed at higher doses of the ADP extract (Fig. 3b). Moreover, ADP extract was tested to ascertain DNA fragmentation in HCC cells. The results obtained (Fig. 3c) showed undamaged DNA with the intact band in control well, whereas treated cells displayed progressive DNA fragmentation in a dose-dependent manner. Little DNA shearing was observed at 15 mg/mL of extract which was found to increase at 20 mg/mL of ADP extract.

### Annexin V-FITC double stain revealed ADP extract mediated induction of apoptosis in HepG2 cells

**Figure 4:**
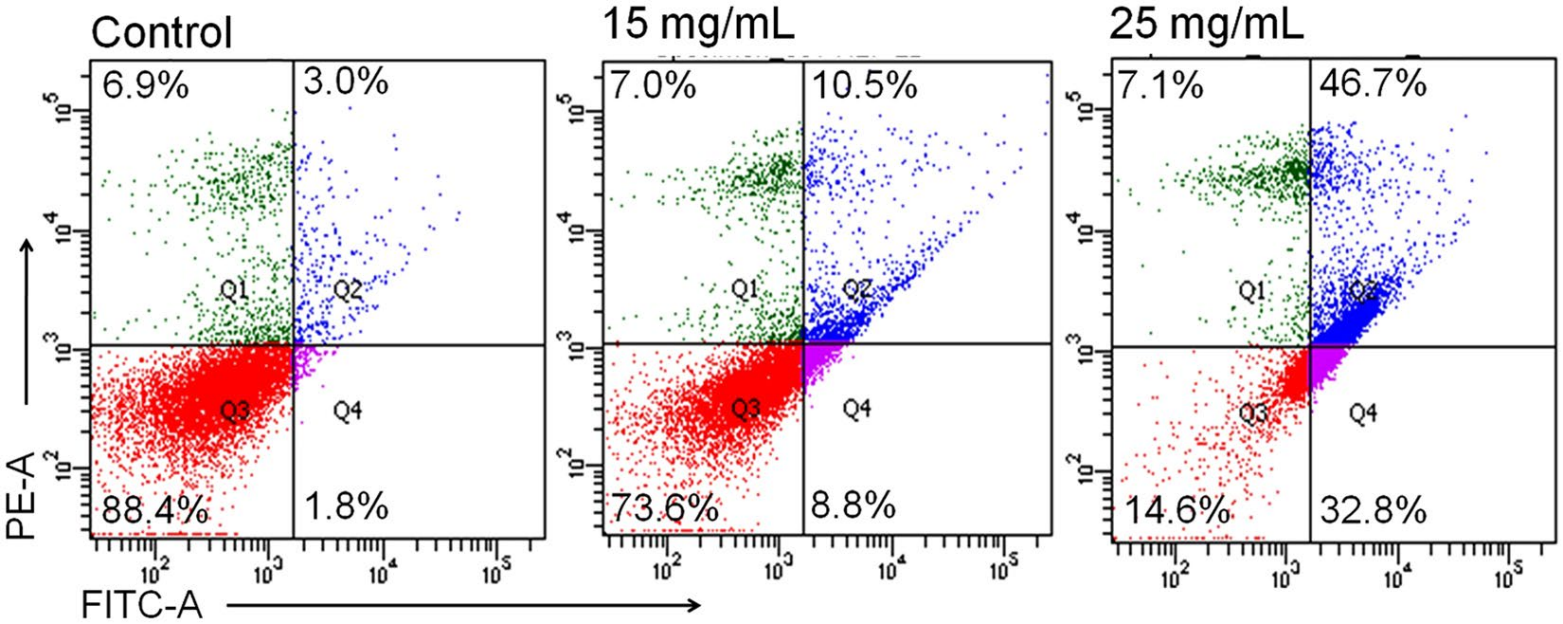
ADP extract mediated induction of apoptosis in human HCC cells. Flow cytometry analysis of HCC cells after 48 h treatment period with 15 and 25 mg/mL of ADP extract. Representative figures showing the population of viable (annexin V^−^ PI^−^), early apoptotic (annexin V^+^ PI^−^), late apoptotic (annexin V^+^ PI^+^) and necrotic (annexin V^−^ PI^+^) cells.

To confirm the quantitative efficacy of apoptosis induction, HCC cells were evaluated further by Annexin V-FITC Apoptosis Detection Kit (Biovision, USA). Untreated cells showed 88.4% viability and were typed as being alive and healthy whereas 15 mg/mL ADP extract increased the cell death by inducing 8.8% early apoptotic and 10.5% late apoptotic cells (Fig. 4). Moreover, 25 mg/mL ADP extract caused induction of a remarkable 32.8% early apoptotic and 46.7% late apoptotic cells as compared to control group.

### ADP extract induced intracellular ROS generation

**Figure 5:**
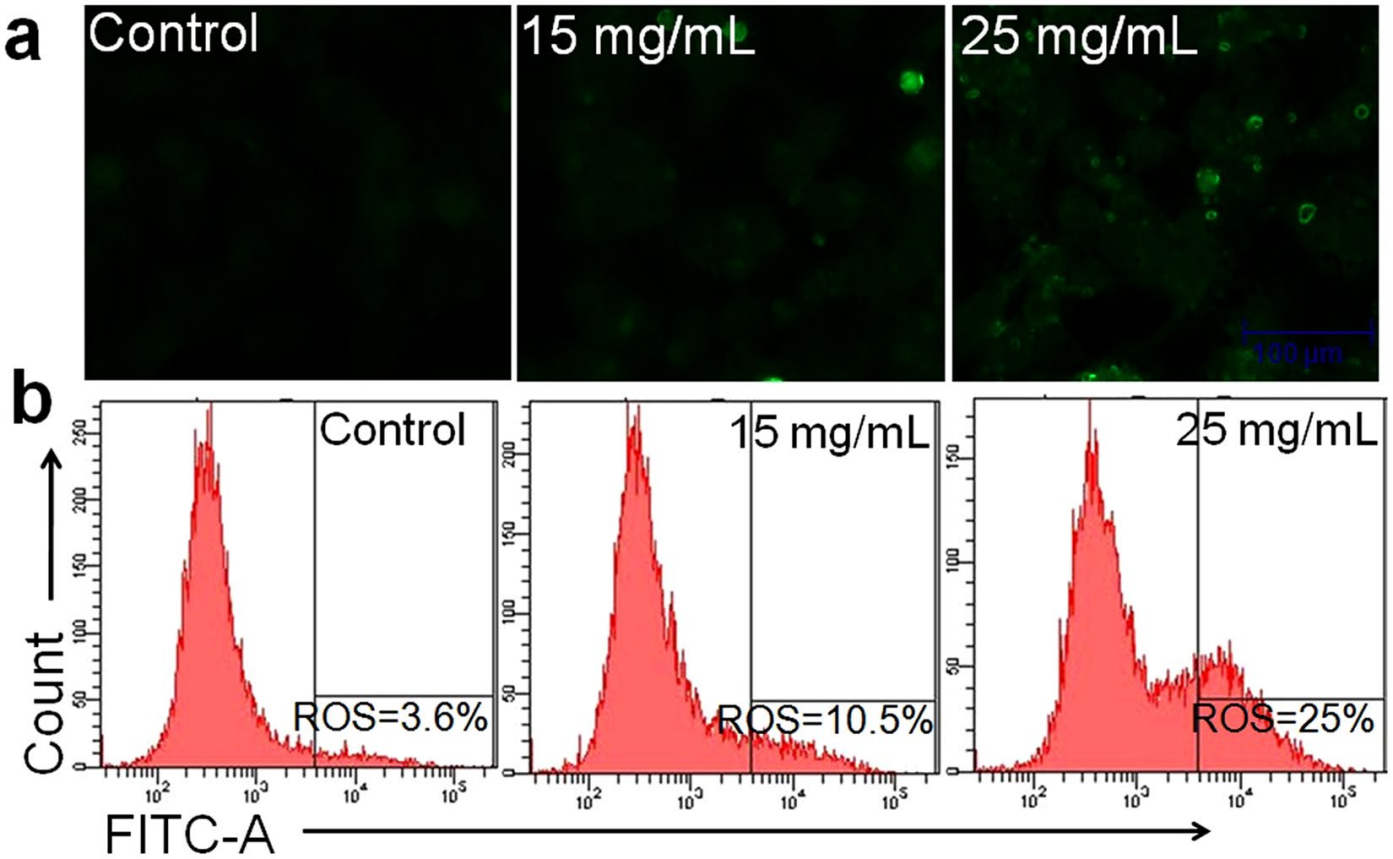
Intracellular ROS generation in human HCC cells induced by different concentrations of ADP extract (**a**) Photomicrographs showing intracellular ROS generation induced by two effective concentrations (15 and 25 mg/mL) of ADP extract after 12 h incubation. Photomicrographs were taken with a fluorescence microscope. Scale bar = 100 μm (**b**) The fluorescence in the cells is represented as the percentage of ROS production analyzed using flow cytometry.

As revealed in Fig. 5a, HCC cells treated with ADP extract showed a significant increase in ROS intensity in a dose-dependent manner as compared to untreated cells. The results of the flow cytometry measurement of ROS generation showed that control cells displayed 3.6% ROS which is a characteristic of normal healthy cells that generate little amount of ROS intensity. However, 15 mg/mL of ADP extract enhanced ROS level by 10.5% as compared to control. Moreover, ROS production was found to increase enormously by 25% at 25 mg/mL of ADP extract (Fig. 5b).

### ADP extract decreased the MMP

**Figure 6:**
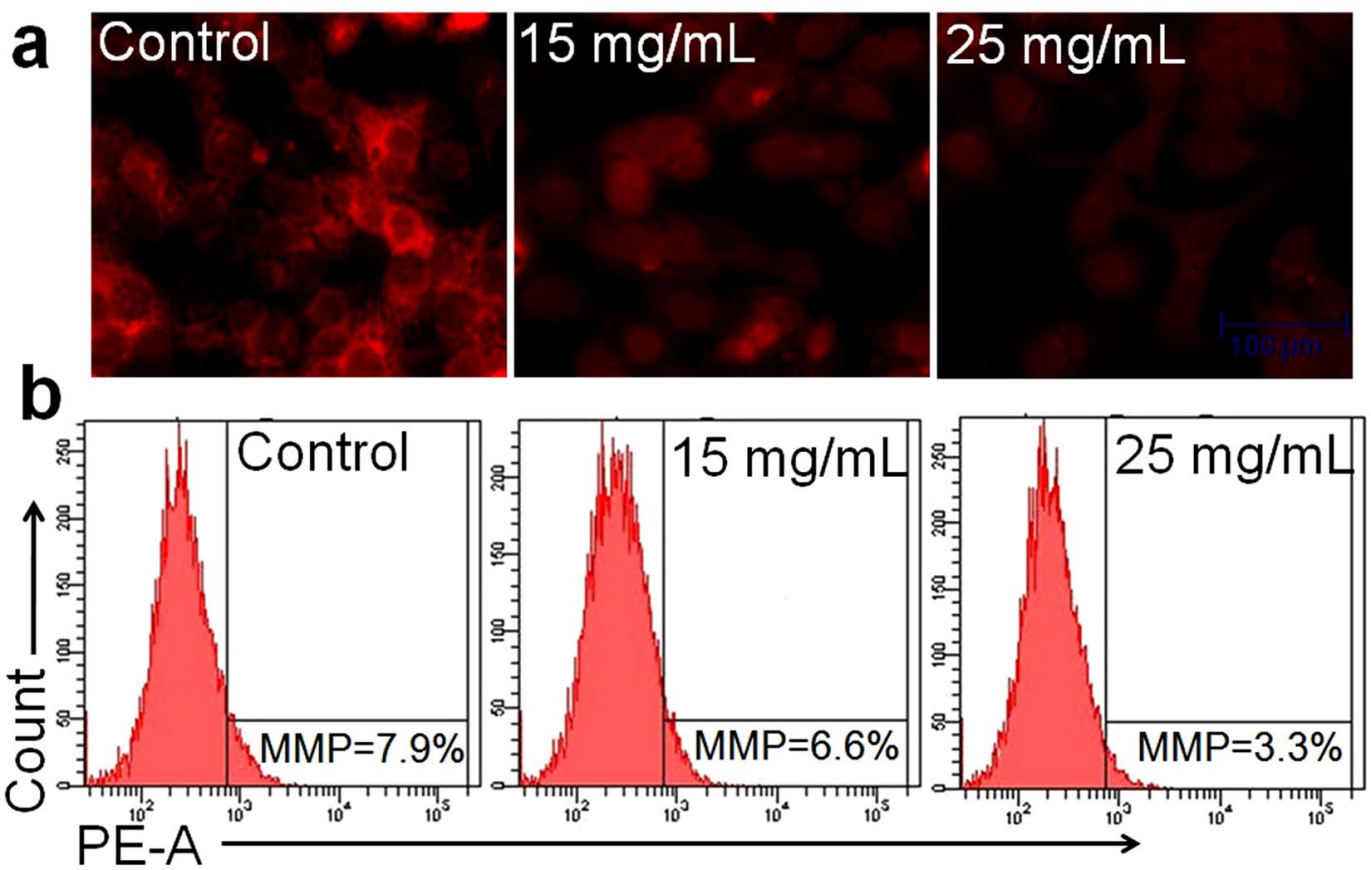
Mitochondrial membrane potential of human HCC cells stained with Rh 123 dye. (**a**) Photographs indicate a decrease in MMP (an early event in apoptosis) with increasing concentrations of ADP extract. Photomicrographs were taken with a fluorescence microscope. Scale bar = 100 μm. (**b**) Fluorescence in the cells is represented as the percentage of MMP reduction in HepG2 cells analyzed by flow cytometry.

Loss of MMP was indicated by the decrease of red fluorescence of the fluorescent dye Rh 123 as revealed in the photomicrograph (Fig. 6a). Figure 6b depicts the percent MMP activity analyzed by flow cytometry. Results suggested that treatment of HepG2 cells with ADP extract resulted in a dose-dependent decrease in the percent MMP level in treated HCC cells. The untreated cancer cells exhibited 7.9% MMP whereas it was decreased to 6.6% and 3.3% at 15 and 25 mg/mL of ADP extract, respectively.

### ADP extract induced S and G2/M phase arrest

**Figure 7:**
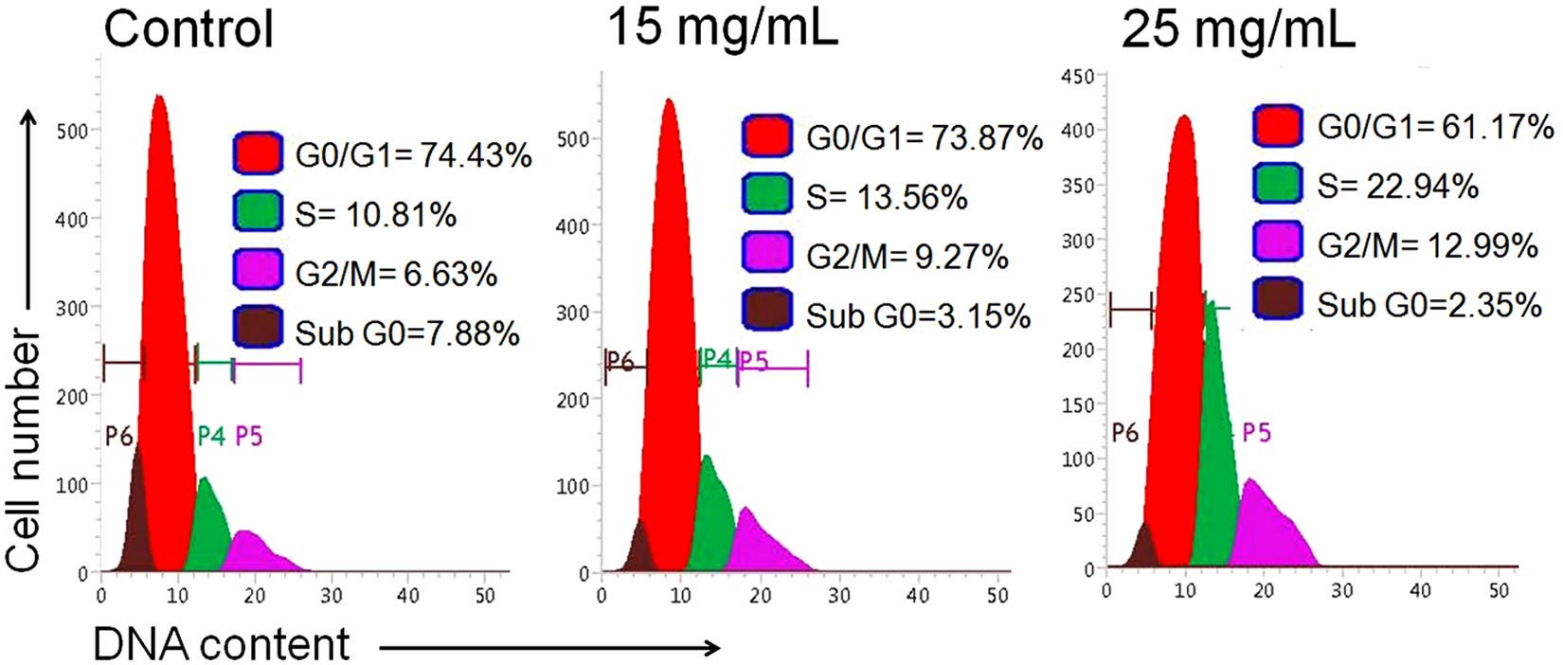
DNA content analysis by flow cytometry. Pictorial graph showing the mean proportion of cells in different phases of cell cycle treated with 15 and 25 mg/mL of ADP extract after 48 h.

HCC cells were treated with ADP extract for 24 h and then subjected to cell cycle analysis using flow cytometry. As shown in Fig. 7, ADP extract substantially increased the number of HCC cells in S phase, which was accompanied by a proportional decrease in the percentage of cells in the G0/G1 phase. ADP extract also sparingly increased the proportion of cells in the G2/M phase of the cell cycle. These results indicate that ADP arrestedHCC cells in both S and G2/M phase of the cell cycle.

### ADP extract regulated the apoptosis-related genes in HCC cells

**Figure 8:**
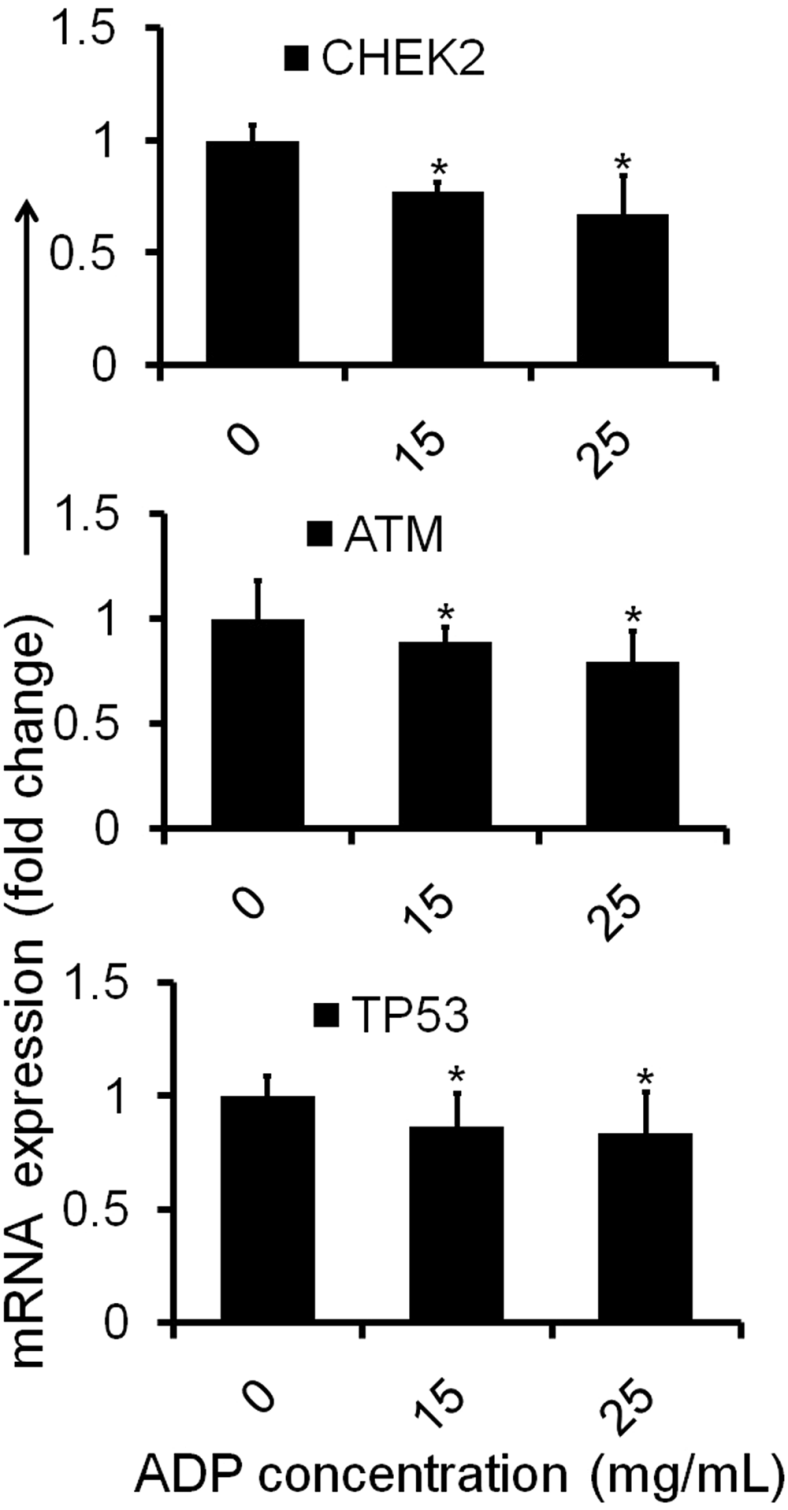
Molecular profiling by measurement of mRNA levels of apoptosis-associated genes. The HCC cells were treated with 15 and 25 mg/mL of ADP extract for 48 h and subjected to qRT-PCR analysis. Bar graph represents the expression profiles of CHEK2, ATM and TP53 genes. Data are represented as mean ± SEM (n = 3). ^*^p < 0.05 significant difference to control.

ADP extract was further used to investigate the expression of tumor suppressor genes of TP53 pathway and to ascertain whether these were involved in the apoptosis-inducing mechanism of cell death or not. As is clear from the qRT-PCR analysis, ADP extract modulated the expression of tumor suppressor genes such as CHEK2, ATM and TP53 in treated HCC cells. As shown in Fig. 8, ADP extract decreased the expression of CHEK2 by 0.77-fold and 0.67- fold at 15 and 25 mg/mL, respectively, as compared to control. Likewise, ADP extract decreased the expression of ATM gene by 0.88 and 0.79 fold at 15 and 25 mg/mL, respectively, when compared with the control group. The TP53 gene also showed a slight downregulation in its expression level as compared to untreated control group. These results suggested that ADP extract did not change the expression of tumor suppressor genes of the TP53 pathway in HCC cells significantly as compared to the control.

## Discussion

The present study undertook the unprecedented investigation of cytotoxicity and underlying mechanism of apoptotic cell death induced by ADP extract on human HCC HepG2 cells. This study also examined the active component of ADP extract, which may have a potential role in causing cytotoxicity. The result of the HPLC study revealed the presence of β-D-glucan as an active component in ethanolic extract of ADP. A previous study has reported that β-glucan, a phytochemical component of Libyan Ajwa dates, demonstrated antitumor activity against solid tumor in mice, which was found to be related to (1 → 3)-β-D-glucan linkages^24^. Likewise, a study has examined the anticancer activity of the low molecular weight β-glucan from oats against skin melanoma cell line Me45 and skin epidermoid carcinoma cell line A431, which significantly decreased cancer cell viability in a time- and dose-dependent manner, while for the normal keratinocyte cell line HaCaT, it was reported to be non-toxic^25^. Interestingly, the present study also demonstrated that ADP extract mediated both dose- and time-dependent anti-proliferative effects against HCC cells, while having no toxicity against the normal Vero cell line. A previous study has reported that the date pulp also contains phenolics like quercetin and kaempferol^15^, which possess anticancer activity against HCC cells^26,27^. Thus, it can be postulated that the anticancer effect of ADP extract against HCC cells might be due to the synergistic or combined effect of the potential bioactive components of Ajwa dates.

Previously, various plant extracts have been evaluated for their anticancer activity against HCC cell line. The IC_50_ values of Chinese medicine Fanbaicao extract and aqueous extract of *Chlorella vulgaris *have been reported to be 2.03 and 1.6 mg/mL, respectively, at 24 h^28,29^, whereas 1.30 mg/mL for *Sophora moorcroftiana* seed extract at 48 h^30^. In the present study, ADP extract induced cell death of HCC cells with an IC_50_ value of 20.03 and 16.78 mg/mL after 24 and 48 h exposure, respectively (Fig. 2d). In a previous study, the total sugar content of Ajwa date pulp was found to be 74.3 g/100 g dry weight^31^. Because of a large amount of polysaccharide in ADP, higher doses would be needed for the efficacy of ADP extract against HCC cells. Interestingly, Al-Bukhaari (5445) and Muslim (2047) have narrated from Sa’d ibn Abi Waqqaas that the Prophet (PBUH) said: “Whoever eats seven Ajwa dates in the morning, will not be harmed by any poison or witchcraft that day.” This is the reason why one should intake a large amount of Ajwa pulp to lead a healthy life.

Previous studies have also reported the growth inhibitory and cytotoxic effects of ADP extract against various cancer cells^18,19^. The cell viability data of the present study has indicated that ADP extract is equally effective on HCC cells. However, the underlying mechanism of ADP extract mediated anti-proliferative effect in HCC cells has not been studied so far.

The morphological data revealed that ADP extract treated HCC cells acquired a round shape, showed cluster shrinkage and detachment from the surface. In contrast, untreated cells remained intact with regularity in shape. This result showed the initial characteristic features of apoptotic cell death^32^. To confirm the efficacy of apoptosis, this study further investigated the major apoptotic events in ADP extract treated HCC cells under a fluorescence microscope. Nuclear condensation data revealed that exposed cells displayed typical apoptotic features *viz*. chromatin condensation, fragmented nuclei and extensive cytoplasmic vacuolization as compared to untreated cells (Fig. 3). The AO/EtBr double stain data depicted early and late apoptosis in ADP treated cells. The early apoptotic cells were detected *via* the binding of AO within the fragmented DNA displaying a bright green fluorescence at a low dose of ADP extract. However, higher dose of ADP extract led to the late stages of apoptosis as indicated by the presence of a reddish-orange color because of the binding of PI to denatured DNA. Moreover, to justify these results quantitatively, a flow cytometry analysis of Annexin-V/PI double stain was performed. The result indicated that the percentage of viable cells was decreased with a concomitant increase in the percentage of cells undergoing early and late apoptosis. A lower dose of the ADP extract led to early apoptotic cells while late apoptotic stages were found at a higher dose of the ADP extract (Fig. 4). This quantitative data suggested that ADP extract prompted most of the cells into late apoptosis stage and induced cancer cell death. A previous study has also reported that methanolic extract of Ajwa dates induced apoptosis in breast cancer MCF-7 cells by increasing the percentage of cells in late apoptotic stage^18^. DNA fragmentation data also confirmed the apoptotic efficacy of ADP extract against HCC cells.

To confirm the apoptotic mechanism of cell death, intracellular ROS generation was evaluated in ADP treated HCC cells. Overproduction of ROS disrupts the plasma membrane and cytoskeleton and finally leads to chromosomal damage^33^. ROS has been regarded as an important regulator of both extrinsic and intrinsic pathways of cell survival and cell death^34^. Various natural agents that are used as anticancer compounds can lead to cell death of many cancer cells by causing overproduction of ROS^35^. Flow cytometry analysis of ROS generation confirmed that ADP extract stimulated ROS production in HCC cells by causing oxidative stress, destabilizing mitochondria and consequently induced apoptosis (Fig. 5).

Mitochondria play a vital role in both cell survival and cell death by sending the death signals to the cascades. When cells undergo apoptosis, the mitochondria lose their membrane integrity and release cytochrome c into the cytosol that ultimately leads to the formation of apoptosome and completes the intrinsic apoptotic pathway^36,37^. In the present study, both fluorescence microscopy and flow cytometry data showed the disruption of the mitochondrial membrane integrity and loss of MMP in ADP extract treated HCC cells (Fig. 6). Loss of fluorescence intensity of Rh 123 dye inside mitochondria due to loss of mitochondrial integrity revealed the comprehensible difference between the apoptotic and viable cells. This study suggested that ADP extract induced the apoptotic events through the intrinsic pathway.

Cell-cycle arrest in response to stress is integral to the maintenance of genomic integrity. Cell cycle arrest provides sufficient time for the cells to repair damaged DNA. In case of severe damage, cells proceed to apoptosis, thus stopping the proliferation of cancer cells^38^. The cell cycle analysis in the present study revealed a higher percentage of cells in the S and G2/M phase whereas the percentage of cells in the G0/G1 phase was decreased as compared to control cells (Fig. 7). These findings are consistent with a previously published study in which paclitaxel, an anticancer drug, inhibited human tenon’s fibroblast cell proliferation through cell cycle arrest at both S and G2/M phases^39^. These results indicated that ADP extract inhibited cell proliferation *via* S and G2/M phase arrest in a dose-dependent manner.

The present study has also attempted a validation of our hypothesis about TP53 implication in the ADP-extract mediated apoptosis of HCC cells. For this study, the gene expression of ATM, CHEK2 and TP53 were analyzed by qRT-PCR. The ATM is a key checkpoint molecule regulating cell cycle responses to DNA damage either by cell cycle arrest or apoptosis^40^. CHEK2, a tumor suppressor gene, is involved in DNA repair, cell cycle arrest or apoptosis in response to DNA damage^41^. Upon severe damage of cells, ATM along with ataxia telangiectasia and Rad3-related protein (ATR) phosphorylates and activates the protein kinase CHEK2 which results in the activation of tumor suppressor gene TP53^42^. Increased level of TP53 augments numerous target genes such as p21, Mdm2 and Bax that mediate cell cycle arrest and apoptotic cell death^43^. On the basis of cell cycle arrest, other possible mechanisms of cell death in HCC cells can be suggested. In a previous study, pemetrexed induced S-phase arrest and apoptosis in human non-small-cell lung cancer A549 and H1299 cell lines through serine/threonine protein kinase (Akt) activation which stimulated Cdk2/Cyclin A-associated kinase activation and then promoted the movement of cells into the S phase^44^. Another recent study has shown that the down-regulation of serine-threonine kinase receptor-associated protein (STRAP), an important regulator of cell proliferation might be responsible for the anti-proliferation and S-phase arrest in HepG2 cells by blocking the DNA repair function of p53^30^. On the basis of G2/M phase arrest, another possible mechanism of apoptotic cell death may be implicated. A study has shown that inhibition of c-Jun N-terminal kinase (JNK) leads to a G2/M phase arrest in breast cancer cells independent of p53 function^45^. Moreover, a previous report showed that erianin induced apoptosis and G2/M-phase arrest in human osteosarcoma cells *via* the ROS/JNK signaling pathway^46^. Thus, it can be postulated that ADP extract induced apoptotic cell death in HCC might be mediated through the Akt pathway, downregulation of STRAP as well as through ROS/JNK signaling pathway. The present study, thus, confirmed that ADP extract mediated cell cycle arrest at both S and G2/M phase in HCC cells followed by apoptosis through a TP53-independent pathway. Interestingly, in independent studies, Artonin E, a ruthenium- xanthoxylin complex and a novel analog of varacin C have been reported to induce apoptosis in target cancer cells *via* a TP53-independent pathway^47–49^. It would be our endeavor in future to evaluate and assess the anticancer activity of ADP-extract *via* alternative pathways of apoptosis induction *in vitro* and *in vivo*.

In conclusion, the present study revealed the potent growth-inhibitory effect of ethanolic extract of ADP against human liver carcinoma HepG2 cells with little to no effect on normal Vero cells. The effect was found to be associated with ROS generation and MMP depletion in cancer cells. Moreover, ADP extract induced DNA damage in HCC cells leading to cell cycle arrest at S and G2/M phases, and followed by apoptosis through a TP53-independent pathway. This study also examined the presence of β-D-glucan in ADP extract, which has a potential role in apoptotic cell death. To conclude, ADP extract has the potential for development into a novel and potent anticancer drug against human liver cancer in future, albeit with further clinical studies to validate the therapeutic basis of drug development.

## Electronic supplementary material

Supplementary Information

## References

[1] Kanavos P (2006). The rising burden of cancer in the developing world. Ann. Oncol..

[2] Pacheco, S. O. *et al*. Food Habits, Lifestyle Factors, and Risk of Prostate Cancer in Central Argentina: A Case Control Study Involving Self-Motivated Health Behavior Modifications after Diagnosis. *Nutrients* **8**, 10.3390/nu8070419 (2016). 10.3390/nu8070419 PMC4963895 27409631

[3] Zhu RX, Seto WK, Lai CL, Yuen MF (2016). Epidemiology of Hepatocellular Carcinoma in the Asia-Pacific Region. Gut Liver.

[4] Akinyemiju T (2015). The Burden of Primary Liver Cancer and Underlying Etiologies From 1990 to 2015 at the Global, Regional, and National Level: Results From the Global Burden of Disease Study. JAMA oncol..

[5] Yang JD, Roberts LR (2010). Hepatocellular carcinoma: A global view. Nat. Rev. Gastroenterol. Hepatol..

[6] Dimitroulis D (2017). From diagnosis to treatment of hepatocellular carcinoma: An epidemic problem for both developed and developing world. World J. Gastroenterol..

[7] Miyahara K, Nouso K, Yamamoto K (2014). Chemotherapy for advanced hepatocellular carcinoma in the sorafenib age. World J. Gastroenterol..

[8] Gonzales GF, Valerio LG (2006). Medicinal plants from Peru: a review of plants as potential agents against cancer. Anticancer Agents Med. Chem..

[9] Cragg GM, Grothaus PG, Newman DJ (2009). Impact of natural products on developing new anti-cancer agents. Chem. Rev..

[10] Li Y, Martin RC (2011). Herbal medicine and hepatocellular carcinoma: applications and challenges. Evid. Based Complement. Alternat. Med..

[11] Mallhi TH (2014). Review: Ajwa date (Phoenix dactylifera)- an emerging plant in pharmacological research. Pak. J. Pharm. Sci..

[12] Hasan M, Mohieldein A (2016). *In Vivo* Evaluation of Anti Diabetic, Hypolipidemic, Antioxidative Activities of Saudi Date Seed Extract on Streptozotocin Induced Diabetic Rats. J. Clin. Diagn. Res..

[13] Al-Farsi MA, Lee CY (2008). Nutritional and functional properties of dates: a review. Crit. Rev. Food Sci. Nutr..

[14] Nasir MU (2015). A review on the nutritional content, functional properties and medicinal potential of dates. Sci. Lett..

[15] Abdul-Hamid NA (2018). Metabolite characterization of different palm date varieties and the correlation with their NO inhibitory activity, texture and sweetness. J Food Sci Technol..

[16] Taleb H, Maddocks SE, Morris RK, Kanekanian AD (2016). Chemical characterisation and the anti-inflammatory, anti-angiogenic and antibacterial properties of date fruit (Phoenix dactylifera L.). J. Ethnopharmacol..

[17] Khan F (2017). Anti-cancer effects of Ajwa dates (Phoenix dactylifera L.) in diethylnitrosamine induced hepatocellular carcinoma in Wistar rats. BMC Complement. Altern. Med..

[18] Khan F (2016). Ajwa Date (Phoenix dactylifera L.) Extract Inhibits Human Breast Adenocarcinoma (MCF7) Cells *In Vitro* by Inducing Apoptosis and Cell Cycle Arrest. PLoS One.

[19] Mirza MB (2018). Induction of apoptosis and cell cycle arrest by ethyl acetate fraction of Phoenix dactylifera L. (Ajwa dates) in prostate cancer cells. J. Ethnopharmacol..

[20] Siddiqui S (2015). Cissus quadrangularis Linn exerts dose-dependent biphasic effects: osteogenic and anti-proliferative, through modulating ROS, cell cycle and Runx2 gene expression in primary rat osteoblasts. Cell prolif..

[21] Khan MA, Ahmad R, Srivastava AN (2017). Effect of ethyl acetate aroma on viability of human breast cancer and normal kidney epithelial cells *in vitro*. Integr. Med. Res..

[22] Husain I (2018). Phytochemical characterization and biological activity evaluation of ethanolic extract of Cinnamomum zeylanicum. J. Ethnopharmacol..

[23] Fan J (2015). Induction of mitochondrial dependent apoptosis in human leukemia K562 cells by meconopsis integrifolia: A species from traditional Tibetan medicine. Molecules.

[24] Ishurd O, Kennedy JF (2005). Anticancer Activity of Polysacharides prepared from Libyan Dates (Phoenix dactylifera L.). Carbohydr. Polym..

[25] Choromanska A (2015). Anticancer properties of low molecular weight oat beta-glucan – An *in vitro* study. Int J Biol Macromol..

[26] Zhou J (2017). Investigation of the anti-cancer effect of quercetin on HepG2 cells *in vivo*. PLoS One.

[27] Guo H (2017). Kaempferol induces hepatocellular carcinoma cell death via endoplasmic reticulum stress-CHOP-autophagy signaling pathway. Oncotarget.

[28] Liu L (2016). Effect of Fanbaicao (Herba Potentillae Discoloris) oil on the expression of p21 and CDK4 in HepG2cells. J. Tradit. Chin. Med..

[29] Yusof YA, Saad SM, Makpol S, Shamaan NA, Ngah WZ (2010). Hot water extract of Chlorella vulgaris induced DNA damage and apoptosis. Clinics (Sao Paulo).

[30] Su G (2018). Anti-proliferation effects of ethanolic extracts from Sophora moorcroftiana seeds on human hepatocarcinoma HepG2 cell line. Nat. Prod. Res..

[31] Assirey EA (2015). Nutritional composition of fruit of 10 date palm (Phoenix dactylifera L.) cultivars grown in Saudi Arabia. Journal of Taibah University for science..

[32] Taatjes DJ, Sobel BE, Budd RC (2008). Morphological and cytochemical determination of cell death by apoptosis. Histochem. Cell Biol..

[33] Guo C, Sun L, Chen X, Zhang D (2013). Oxidative stress, mitochondrial damage and neurodegenerative diseases. Neural Regen. Res..

[34] Davalli P, Mitic T, Caporali A, Lauriola A, D’Arca D (2016). ROS, cell senescence, and novel molecular mechanisms in aging and age-related diseases. Oxid. Med. Cell Longev..

[35] Lee EB (2017). The quinone-based derivative, HMNQ induces apoptotic and autophagic cell death by modulating reactive oxygen species in cancer cells. Oncotarget.

[36] Jeong SY, Seol DW (2008). The role of mitochondria in apoptosis. BMB rep..

[37] Wang C, Youle RJ (2009). The role of mitochondria in apoptosis. Annu. Rev. Genet..

[38] Vessoni AT, Filippi-Chiela EC, Menck CF, Lenz G (2013). Autophagy and genomic integrity. Cell death differ..

[39] Chen N (2016). Paclitaxel inhibits cell proliferation and collagen lattice contraction via TGF-β signaling pathway in human tenon’s fibroblasts *in vitro*. Eur. J. Pharmacol..

[40] Maréchal A, Zou L (2013). DNA damage sensing by the ATM and ATR kinases. Cold Spring Harb Perspect. Biol..

[41] Zannini L, Delia D, Buscemi G (2014). CHK2 kinase in the DNA damage response and beyond. J. Mol. Cell Biol..

[42] Jiang H (2009). The combined status of ATM and p53 link tumor development with therapeutic response. Genes Dev..

[43] Beckerman R, Prives C (2010). Transcriptional regulation by p53. Cold Spring Harb Perspect. Biol..

[44] Chen KC, Yang TY, Wu CC, Cheng CC, Hsu SL (2014). Pemetrexed induces S-phase arrest and apoptosis via a deregulated activation of Akt signaling pathway. PLoS One.

[45] Mingo-Sion AM, Marietta PM, Koller E, Wolf DM, Van Den Berg CL (2004). Inhibition of JNK reduces G2/M transit independent ofp53, leading to endoreduplication, decreased proliferation, and apoptosis in breast cancer cells. Oncogene.

[46] Wang H, Zhang T, Sun W, Wang Z, Zuo D (2016). Erianin induces G2/M-phase arrest, apoptosis, and autophagy via the ROS/JNK signaling pathway in human osteosarcoma cells *in vitro* and *in vivo*. Cell Death Dis..

[47] Etti IC (2017). Artonin E induces p53-independent G1 cell cycle arrest and apoptosis through ROS-mediated mitochondrial pathway and livin suppression in MCF-7 cells. Drug Des. Devel. Ther..

[48] de Carvalho NC (2018). A novel ruthenium complex with xanthoxylin induces S-phase arrest and causes ERK1/2-mediated apoptosis in HepG2 cells through a p53-independent pathway. Cell Death Dis..

[49] Zhou, J. *et al*. Varacin-1, a novel analog of varacin C, induces p53-independent apoptosis in cancer cells through ROS-mediated reduction of XIAP. *Acta Pharmacol. Sin*., 10.1038/s41401-018-0005-y (2018). 10.1038/s41401-018-0005-y PMC6329757 29773887

