# Supplementary material for: Cytostatic and Anti-tumor Potential of Ajwa Date Pulp against Human Hepatocellular Carcinoma HepG2 Cells
Source: Sci Rep. 2019 Jan 21;9:245. doi: 10.1038/s41598-018-36475-0 (PMC6341075; doi:10.1038/s41598-018-36475-0)
Supplement: Supplementary file 1 — Supplementary Information [file 41598_2018_36475_MOESM1_ESM.docx]

## Cytostatic and Anti-tumor Potential of Ajwa Date Pulp against Human Hepatocellular Carcinoma HepG2 Cells

Sahabjada Siddiqui^1,*^, Rumana Ahmad^2^, Mohsin Ali Khan^3^, Shivbrat Upadhyay^2^, Ishrat Husain^2^, Anand Narain Srivastava^4^

^1^Department of Biotechnology, Era’s Lucknow Medical College & Hospital, Era University, Lucknow-226003, UP, India.

^2^Department of Biochemistry, Era’s Lucknow Medical College & Hospital, Era University, Lucknow-226003, UP, India.

^3^Chancellor, Era University, Lucknow-226003, UP, India

^4^Department of Pathology, Era’s Lucknow Medical College & Hospital, Era University, Lucknow-226003, UP, India

^*^Corresponding Author

S. Siddiqui

Department of Biotechnology, Era’s Lucknow Medical College & Hospital, Era University, Lucknow-226003, India.

Phone: +91-9452688313

Email: sahabjadabiotech04@gmail.com

**Table S1. Peak results of β-D-glucan standard in HPLC chromatogram**

| **S. No.** | **Retention Time** | **Area** | **Height** | **% Area** |
| --- | --- | --- | --- | --- |
|  | 22.659 | 15058 | 411 | 2.73 |
|  | 24.831 | 440560 | 10832 | 79.80 |
|  | 26.592 | 83594 | 1837 | 15.14 |
|  | 29.936 | 12882 | 741 | 2.33 |
